# Supplementary material for: Identification of a binding site for small molecule inhibitors targeting human TRPM4
Source: Nat Commun. 2025 Jan 19;16:833. doi: 10.1038/s41467-025-56131-2 (PMC11743598; doi:10.1038/s41467-025-56131-2)
Supplement: Supplementary file 4 — Source Data [file 41467_2025_56131_MOESM4_ESM.zip › Source Data file/Ekundayo_Trpm4_Source_data[3].docx]

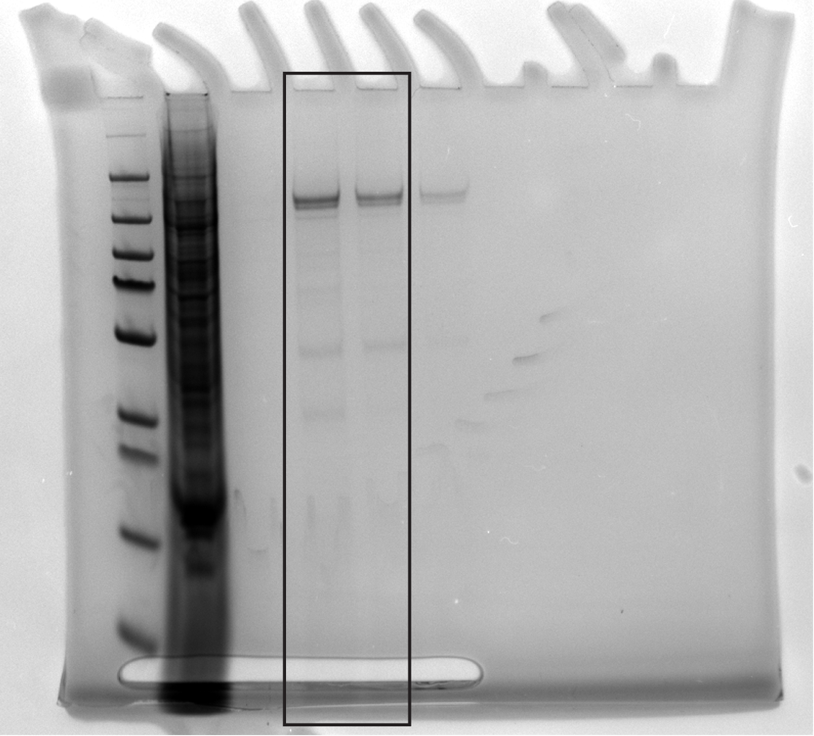


Source data 1 for Supplementary Figure 1b showing SDS-PAGE gel of purified HsTrpm4 following affinity purification. The boxed line indicates the part of the gel shown in the figure.


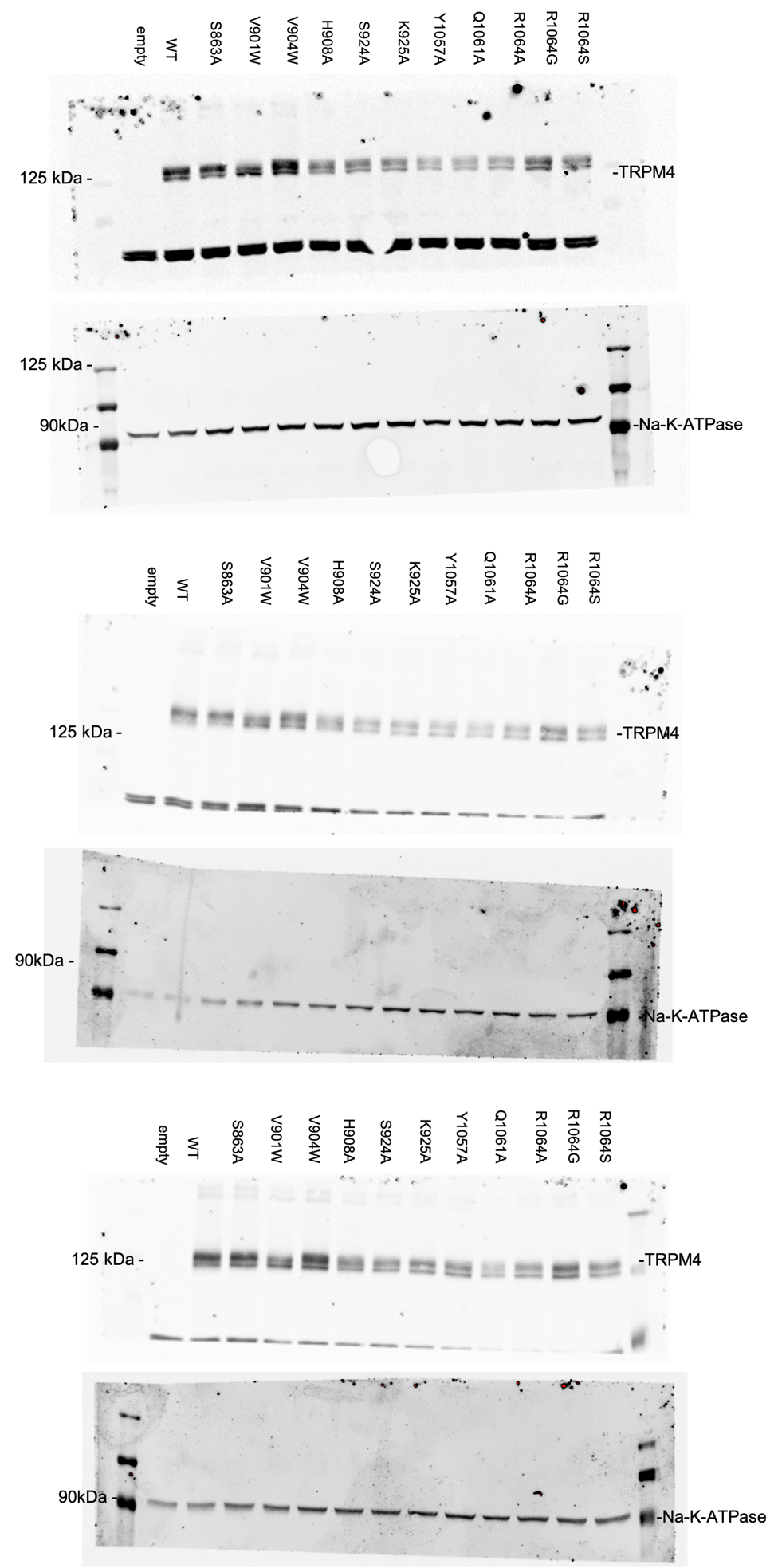


Source data 2 for Supplementary Figure 10a showing the 3 replicates of western blots for the expressin of wildtype (WT) and HsTRPM4 variants expressed in HEK293 cells. Boxed line indicates the region of the gel shown in the figure.


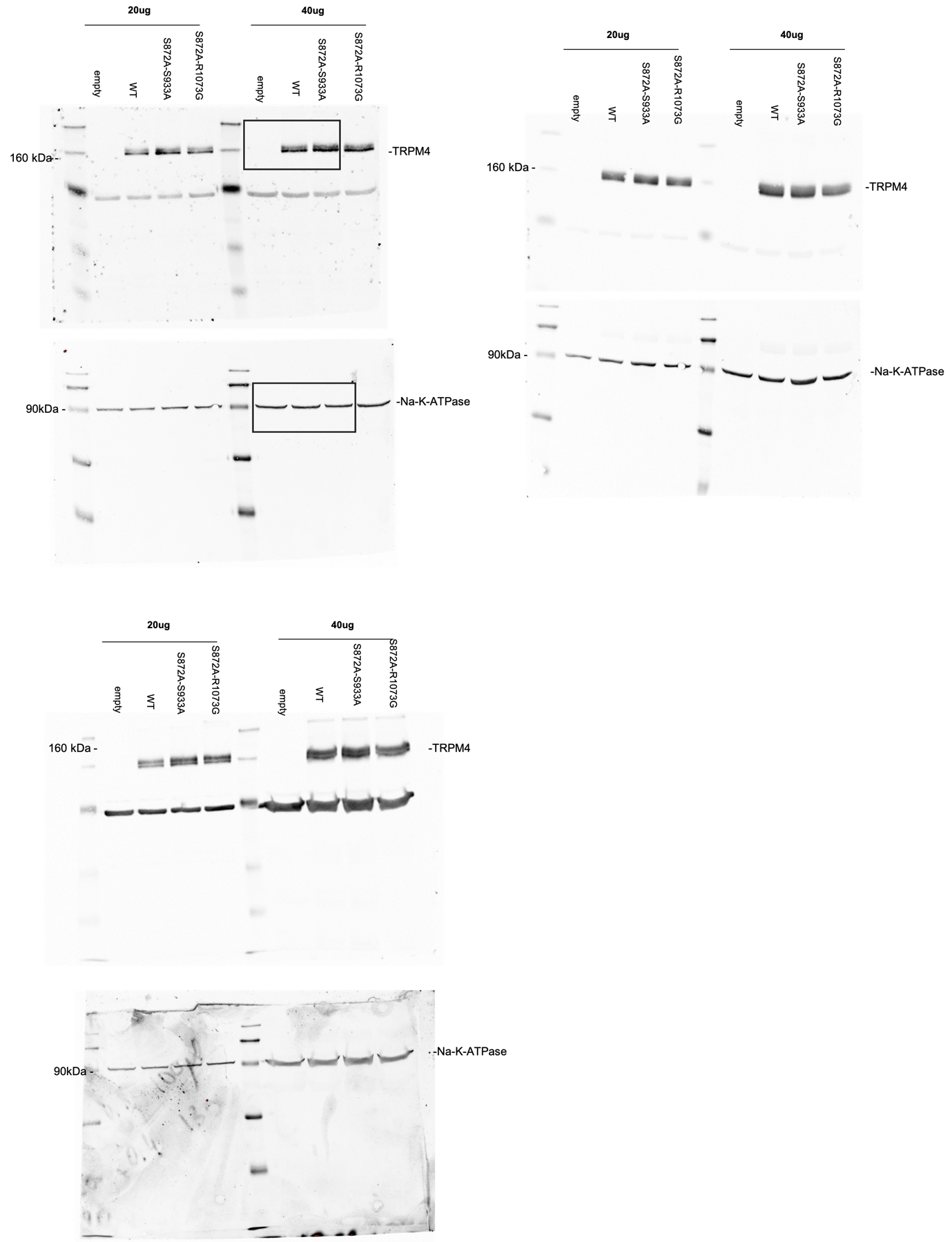


Source data 3 for Supplementary Figure 10a showing the 3 replicates of western blots for the expression of wildtype (WT) and HsTRPM4 double mutation variants expressed in HEK293 cells. Boxed line indicates the region of the gel shown in the figure.
